# Integrated pan-cancer genomic analysis reveals the role of SLC30A5 in the proliferation, metastasis, and prognosis of hepatocellular carcinoma

**DOI:** 10.7150/jca.97214

**Published:** 2024-07-02

**Authors:** Yihan Liu, Tong Lu, Runze Li, Long Cui, Rui Xu, Shenyi Teng, Denis Baranenko, Tianze Zhang, Lida Yang, Rui Qie, Dan Xiao

**Affiliations:** 1 Graduate School, Heilongjiang University of Chinese Medicine, Harbin 150001, Heilongjiang, China.; 2 Medical Technology Department, Qiqihar Medical University, Qiqihar 161006, Heilongjiang, China.; 3 National and Local Joint Engineering Laboratory for Synthesis Transformation and Separation of Extreme Environmental Nutrients, Harbin Institute of Technology, Harbin 150001, Heilongjiang, China.; 4 Department of Oncology, Qiqihar Hospital of Chinese Medicine, Qiqihar 161000, Heilongjiang, China.; 5 National Cancer Center, National Clinical Research Center for Cancer, Cancer Hospital & Shenzhen Hospital, Chinese Academy of Medical Sciences and Peking Union Medical College, Shenzhen, 518116, Guangdong, China.; 6 School of Life Sciences, Faculty of Ecotechnologies, ITMO University, St. Petersburg 197101, Russia.; 7 Department of Thoracic Surgery, The 2nd Affiliated Hospital of Harbin Medical University, Harbin 150081, Heilongjiang, China.; 8 Heilongjiang Nursing Collage, Harbin 150086, Heilongjiang, China.; 9 Department of Geratology, The 1st Affiliated Hospital of Heilongjiang University of Chinese Medicine, Harbin 150001, Heilongjiang, China.; 10 Zhengzhou Research Institute, Harbin Institute of Technology, Zhengzhou 450007, Henan, China.

**Keywords:** SLC30A5, Hepatocellular carcinoma, Prognosis, Proliferation, Migration

## Abstract

**Background:** SLC30A5, a member of the solute transporter protein family, is implicated in tumorigenesis and cancer progression. This study aimed to explore the expression and prognostic significance of SLC30A family genes in pan-cancer, with a specific emphasis on SLC30A5 in hepatocellular carcinoma (HCC).

**Methods:** Expression patterns and prognostic implications of SLC30A family genes were assessed across 33 cancer types, especially HCC. Co-expression analysis explored the relationship between SLC30A5 and immune cell infiltration, immune checkpoints, pathway molecules related to tumor angiogenesis and epithelial-mesenchymal transition (EMT). The role of SLC30A5 in HCC was evaluated through *in vitro* and *in vivo* assays, including CCK8 viability assay, EdU cell proliferation assay, colony formation assay, apoptosis assay, wound healing assay, transwell migration assay, and xenograft mouse model assay using Huh7 cells with targeted knockdown of SLC30A5.

**Results:** SLC30A family genes exhibited overexpression in various tumors. In HCC, upregulation of SLC30A5 expression correlated with adverse prognosis. Significant associations were observed between SLC30A5 expression and immune cell infiltration, immune checkpoints, molecules involved in angiogenesis, and EMT. SLC30A5 overexpression was associated with advanced disease stages, higher histological grades, and vascular invasion. Single-cell RNA sequencing data (GSE112271) revealed notable SLC30A5 expression in malignant cells. *In vitro* and *in vivo* assays demonstrated that SLC30A5 knockdown in Huh7 cells reduced proliferation, migration, and invasion while promoting apoptosis.

**Conclusions:** This study highlights the clinical relevance of SLC30A5 in HCC, emphasizing its role in cell proliferation and migration. SLC30A5 emerges as a promising candidate for a prognostic marker and a potential target in HCC.

## Introduction

Zinc is integral to numerous biological functions in humans, including enzyme regulation, protein structure integrity, DNA synthesis, RNA transcription, and cell proliferation and activation 1, 2. Imbalances in zinc levels are associated with the pathology of various diseases, particularly cancer 3. In this context, zinc transporter proteins, especially those encoded by the SLC30A family genes, are essential for maintaining cellular zinc balance 4. Dysregulation of these proteins is implicated in the initiation and progression of cancer, highlighting their significance in both normal physiological processes and disease pathogenesis.

Previous research has highlighted the significance of individual SLC30A genes in specific cancers, revealing their intricate roles in tumor progression and aggressiveness 5. In breast cancer studies, the absence of SLC30A2 expression in basal-like cells (such as MDA-MB-231, a highly invasive cancer cell) correlates with tumor proliferation and aggressiveness 6, 7. Similarly, in prostate cancer, there is noted upregulation of SLC30A1, SLC30A9, and SLC30A10, alongside downregulation of SLC30A5 and SLC30A6 8. Esophageal cancer exhibits an upregulation of SLC30A7, while colorectal cancer demonstrates a notable increase in the expression of SLC30A5, SLC30A6, and SLC30A7 9. Building on the foundational insights into the varied roles of the SLC30A family across cancer types, this study aims to expand the current understanding through an integrated pan-cancer analysis.

In an endeavor to bridge existing findings and further investigate them, this study employs a multi-omics approach to evaluate the expression of SLC30A family genes and their prognostic significance across various cancers. Leveraging comprehensive datasets from The Cancer Genome Atlas (TCGA) and the International Cancer Genome Consortium (ICGC), this research aims to confirm previous findings and uncover novel insights into the intricate roles of these zinc transporter proteins. The focus is primarily on SLC30A5 within hepatocellular carcinoma (HCC), aiming to enhance the understanding of zinc transporter proteins in cancer progression and to investigate the feasibility of targeting SLC30A5 as a therapeutic intervention for HCC.

## Materials and Methods

### Data Source

RNA sequences, clinicopathological data, and survival data were downloaded from UCSC Xena database (http://xena.ucsc.edu/), TCGA database (http://cancergenome.nih.gov/), and ICGC database (https://dcc.icgc.org/).

### Expression profile analysis

Perl was used to extract the expression data of the SLC30A gene family. The Wilcoxon test was employed to compare expression levels between cancerous and normal tissues. Correlation analysis among the SLC30A genes was conducted using the "corrplot" R package. Expression profiles in various cancer cell lines were assessed using data from the Cancer Cell Line Encyclopedia (CCLE) (https://portals.broadinstitute.org/ccle), with the Kruskal-Wallis test applied for statistical analysis. Protein expression levels across 20 different cancer types were investigated using immunohistochemistry data from the Human Protein Atlas (HPA) (http://www.proteinatlas.org/).

### Genomic alterations and epigenetics analysis

Mutations in the SLC30A gene family were explored using the cBioPortal (http://www.cbioportal.org) for pan-cancer analysis. Methylation patterns were analyzed using the GSCALite web tool (http://bioinfo.life.hust.edu.cn/GSCA/#/), providing insights into the epigenetic regulation of the SLC30A gene family in various cancers.

### Survival analysis

The Kaplan-Meier survival method was implemented using the R packages "survminer" and "survival". The association between gene expression and patient survival was further assessed through forest plots generated by the "survival" and "forestplot" packages. Validation of these findings was conducted using the Kaplan-Meier Plotter (http://kmplot.com/analysis/).

### Correlation with tumor immune infiltration and clinical features

The TIMER 2.0 database (http://timer.comp-genomics.org) was used to assess the correlation between SLC30A5 expression and immune cell infiltration, utilizing diverse methodologies such as TIMER, CIBERSORT, xCell, MCP-counter, EPIC, and quanTIseq. We also analyzed the relationship between SLC30A5 expression and molecules related to tumor angiogenesis and epithelial-mesenchymal transition (EMT), which are known to influence tumor metastasis and malignant proliferation. Additionally, correlations with pathological stages, histological grading, and vascular invasion were analyzed to underline the clinical features of SLC30A5 expression.

### Gene function enrichment analysis

Differential expression analysis was conducted to identify genes associated with high and low SLC30A5 expression levels in HCC samples, using criteria of |logFC| > 1 and FDR < 0.05. The identified differentially expressed genes (DEGs) were subjected to GO and KEGG pathway enrichment analyses using the "ClusterProfiler" package in R. Gene Set Enrichment Analysis (GSEA) was performed with the "enrichplot" package to investigate the biological pathways and functions differentially activated in the SLC30A5 high and low expression groups.

### Cell culture and transfection

The Huh7 cell line was procured from the Institute of Cell Research, Chinese Academy of Sciences (Shanghai, China). Cells were cultured in a humidified incubator set at 37°C with 5% CO_2_. To achieve knockdown of the SLC30A5 gene, Huh7 cells were transduced with pLKO.1 puro lentiviral particles (GeneChem, Shanghai, China). The shRNA sequence employed for this purpose was: 5'-CCGGCCAGATAATTGGATCACTAAACTCGAGTTTAGTGATCCAATTATCTGGTTTTTG-3'. For experimental procedures, the cells were seeded in 96-well plates, facilitating their subsequent infection with the lentiviral particles harboring either shSLC30A5 or a control shRNA. Selection of successfully transfected cells was conducted using 2 µg/mL puromycin over a 48-hour period. The effectiveness of the SLC30A5 knockdown was assessed through RT-PCR and Western blot analysis.

### Real-time PCR

Following lentiviral transfection and the establishment of stable cell lines, total RNA was extracted using TRIzol reagent (Catalog Number: 15596026, Invitrogen; Thermo Fisher Scientific, U.S.A.). The RNA was then reverse-transcribed into cDNA using a reverse transcription kit (Catalog Number: D7170M, Beyotime Biotechnology, China). The resulting cDNA was amplified using PCR, with the Exicycler 96 system (Catalog Number: D7268M, Beyotime Biotechnology, China) employed for the detection of fluorescence. GAPDH served as the internal reference. The primers used were purchased from Sangon Biotech Co., Ltd (Shanghai, China), and the sequences are shown as follows:

SLC30A5: forward: GCCCATCCTGGTTTCTATGC; reverse: GGCTTGATGTTGAACTGCCTTA. GAPDH: forward: TGACTTCAACAGCGACACCCA; reverse: CACCCTGTTGCTGTAGCCAAA.

Relative gene expression levels were calculated using the 2^-ΔΔCq^ method.

### Western blot

Total protein was extracted from Huh7 cells using protein lysis buffer (Sigma, U.S.A.). The proteins were then separated on a 10% SDS-PAGE gel, with each lane loaded with 20 µg of protein, and subsequently transferred onto a PVDF membrane. The membrane was blocked with 5% non-fat milk to prevent non-specific binding and incubated overnight with primary antibodies: anti-SLC30A5 (Catalog Number: 25604-1-AP, Proteintech, Wuhan, China, 1:1000 dilution, Rabbit) and anti-GAPDH (Catalog Number: AF7021, Affinity, 1:3000 dilution, Rabbit) serving as the loading control. This was followed by a 1-hour room temperature incubation with a Goat Anti-Rabbit HRP-conjugated secondary antibody (Catalog Number: S0001, Affinity, 1:3000 dilution). Protein bands were visualized using an ECL chemiluminescence kit and captured with a Syngene gel imaging system (Syngene, Cambridge, U.K.).

### CCK-8 assay

Huh7 cells (2×10^3^ per well) were seeded in 96-well plates and cultured for 24-96 hours under previously outlined conditions. After refreshing the medium, CCK-8 reagent (Catalog Number: C0038, Beyotime Biotechnology, China) was added to each well. Following a 60-minute incubation period, absorbance at 450 nm was measured using a spectrophotometer to evaluate cell viability.

### EdU (5-ethynyl-2'-deoxyuridine) cell proliferation assay

Transfected Huh7 cells (1×10^5^ per well) were seeded in 96-well plates, and supplemented with EdU labeling medium. Subsequent experiments were conducted according to the EdU staining kit protocol (Catalog Number: C0075M, Beyotime Biotechnology, China). Cell nuclei were stained using DAPI (Catalog Number: C1002, Beyotime, Shanghai, China), and images were captured using a fluorescence microscope (FV300, Olympus, Tokyo, Japan).

### Colony formation assay

The cells (1.0×10^3^ per well) were seeded in 6-well plates and incubated in DMEM supplemented with 10% FBS. After 8 days, the cells were fixed with 4% paraformaldehyde (Catalog Number: 10010018, Sinopharm Group, Beijing, China) for 30 minutes. Subsequently, a 0.5% crystal violet solution (Catalog Number: 548-62-9, Sigma, USA) was applied for 15 minutes to stain the cells. The quantification of cell proliferation was achieved by counting the colonies consisting of 50 or more cells, which were considered indicative of positive cell proliferation.

### Wound healing assay

Transfected cells were seeded in 6-well plates. A sterile 200 µL pipette tip was used to create a scratch on the cell monolayer. Serum-free medium was added, and images were taken at 0 and 24 hours.

### Transwell assay

Cells were starved in serum-free medium for 12 hours, then seeded in the upper chamber of a Transwell insert (8 µm pore size, Catalog Number: FTW043-12Ins, Beyotime, Shanghai, China) at a density of 5×10^4^ cells per well. Matrigel was precoated on the insert. The lower chamber was filled with 600µL of medium containing 20% FBS. After 24 hours, cells were fixed with 4% paraformaldehyde, stained with 0.1% crystal violet, and counted manually.

### Apoptosis assay

Cells were cultured in 6-well plates. Apoptosis was evaluated using Annexin V-APC/PI apoptosis detection kit (Catalog Number: AP107, KeyGen Biotech, Nanjing, China) in accordance with the manufacturer's guidelines. Analysis was conducted using flow cytometer (eBioscience, California, USA) to quantify apoptotic cells.

### Xenograft mouse model assay

BALB/c nude mice (male, 6 weeks old) were purchased from the Institute for Experimental Animals of the Chinese Academy of Medical Sciences (Beijing, China). Huh7 cells (5.0×10^6^) with sh-NC or sh-SLC30A5 were subcutaneously inoculated into the shoulder. The nude mice were housed in a standard animal room (25 °C, 12-h light/12-h dark cycle). 22 days post-inoculation, the mice were euthanized, and tumors were collected. This study was approved by the Ethics Committee of Harbin Institute of Technology.

## Results

### Differential expression and correlation analysis of SLC30A family genes with multiple cancers

**Figure 1:**
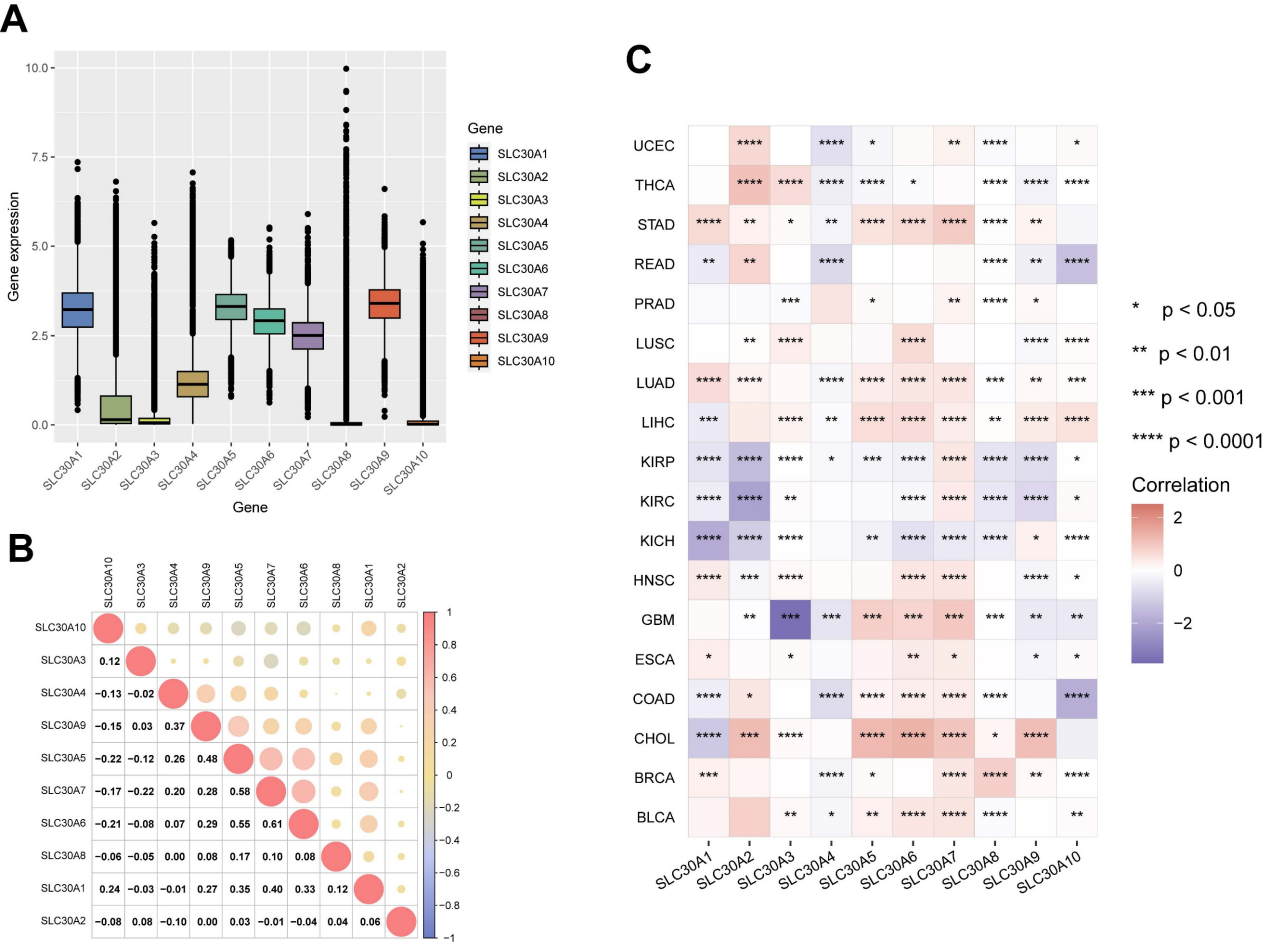
** Expression levels of SLC30A family genes in different tumor types and their correlation. (A)** mRNA expression levels of SLC30A family genes in pan-cancer;** (B)** Correlation analysis among SLC30A gene family members; **(C)** Heatmap representation of log_2_-transformed fold changes illustrating variations in SLC30A family genes expression between cancerous and normal tissues. Statistical significance is denoted as follows: *p<0.05, **p<0.01, ***p<0.001, and ****p<0.0001.

In this study, the mRNA expression levels of the SLC30A family genes across various cancer types within the TCGA database were analyzed. The results revealed relatively high expression of SLC30A1, SLC30A5, SLC30A6, SLC30A7, and SLC30A9 in pan-cancer tissues (Figure 1A). Subsequent correlation analysis indicated potential synergistic interactions within the family, particularly between SLC30A5 and SLC30A6 (correlation coefficient of 0.55), SLC30A6 and SLC30A7 (correlation coefficient of 0.61), and SLC30A5 and SLC30A7 (correlation coefficient of 0.58) (Figure 1B).

Furthermore, the research underscores significant variations in SLC30A gene expression among various cancer types (Figure 1C and Supplementary Figure S1). Specifically, pronounced downregulation of SLC30A genes was observed in urological cancers (KIRP, KIRC, KICH). In contrast, elevation of these genes was noted in liver cancer (LIHC, CHOL), lung cancer (LUAD, LUSC), and breast cancer (BRCA). These findings align with previous reports indicating overexpression of SLC30A2 and SLC30A9 in lung and prostate cancers, as well as heightened SLC30A9 levels in HCC tissues 3, 10.

At the cellular level, overexpression of SLC30A1, SLC30A4, SLC30A5, SLC30A6, SLC30A7, and SLC30A9 was observed in tumor cell lines derived from the central nervous system (CNS), lung, and liver (Supplementary Figure S2). Protein expression analysis confirmed the presence of SLC30A5, SLC30A6, and SLC30A9 in various tumor types, with SLC30A9 consistently demonstrating moderate to high expression across all examined cases. Specifically, elevated levels of SLC30A5 and SLC30A6 were notably observed in pancreatic cancer (Supplementary Table S1). This finding aligns with previous studies reporting elevated levels of SLC30A6 in pancreatic tumors and suggesting that targeting SLC30A6 might potentially inhibit the proliferation of pancreatic cancer cells 11.

### Genomic alterations and epigenetics profiles of SLC30A family genes in pan-cancer

**Figure 2:**
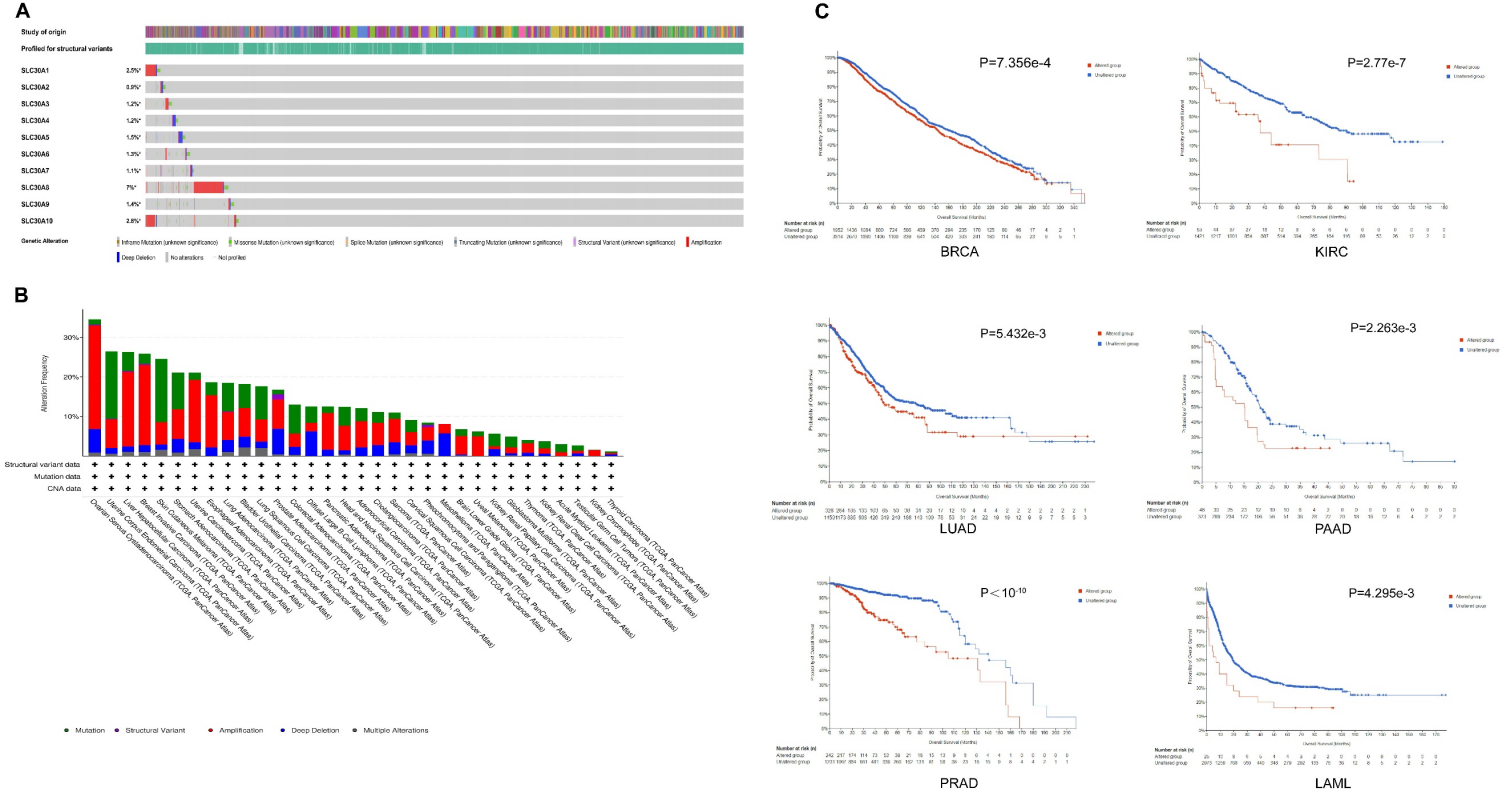
** Genomic alterations in SLC30A family genes in pan-cancer. (A)** Overview of SLC30A genetic alterations across TCGA pan-cancer atlas studies; **(B)** Frequency of alterations in SLC30A genes across various cancer types; **(C)** Correlation between genomic mutations in SLC30A genes and overall survival (OS) in different cancers.

Based on the cBioPortal database, the results indicated that SLC30A8 exhibited a high mutation frequency of approximately 7%, while SLC30A2 had a lower incidence at around 0.9%. Primary genetic alterations observed included amplifications, missense mutations, and deep deletions (Figure 2A). Mutations within the SLC30A family genes were notably frequent across various cancer types, particularly in gynecological (OV, UCEC, UCS), gastrointestinal (STAD, LIHC), breast, and melanoma tumors, with mutation frequencies exceeded 20% (Figure 2B). These genetic alterations were associated with overall survival (OS) in several cancers, including BRCA, KIRC, LUAD, PAAD, PRAD, and LAML (Figure 2C).

Additionally, analysis of methylation patterns of the SLC30A family genes revealed elevated methylation levels in colorectal (COAD), breast (BRCA), kidney (KIRP, KIRC) cancers, contrasted with reduced methylation in lung squamous cell carcinoma (LUSC), bladder cancer (BLCA), and hepatocellular carcinoma (LIHC) (Supplementary Figure S3). Subsequent analysis indicated an inverse relationship between methylation status and mRNA expression within the SLC30A gene family (Supplementary Figure S4).

### Prognostic significance of SLC30A family genes in pan-cancer

Kaplan-Meier curves (Supplementary Figure S5) and Cox regression models (Supplementary Figure S6) were utilized to depict the association between SLC30A family genes and overall survival in pan-cancer. The results indicated that high expression levels of SLC30A genes were associated with lower survival rates in several cancers, particularly in pancreatic cancer and HCC. Specifically, high expression of SLC30A1 in pancreatic cancer was linked to reduced survival, consistent with previous findings regarding its role in pancreatic cancer proliferation and modulation of key pathways such as ERK1/2, p38 MAPK, NF-κB, and mTOR 11. SLC30A5 was significantly associated with the prognosis of multiple cancers, including breast, lung, pancreatic, and liver cancers. Notably, the upregulation of SLC30A5 correlated with a poorer prognosis in HCC, a finding validated by the Kaplan-Meier Plotter (Table 1).

### SLC30A5 as a clinical biomarker for hepatocellular carcinoma

The pan-cancer analysis of the SLC30A family genes provided their expression, genetic alterations, and methylation patterns, highlighting their prognostic significance across various cancers. Notably, the pronounced overexpression of SLC30A5 in HCC drew considerable attention. Its association with adverse clinical outcomes, coupled with genetic and methylation alterations, warranted a deeper investigation to ascertain its prognostic implications in clinical settings.

**Figure 3:**
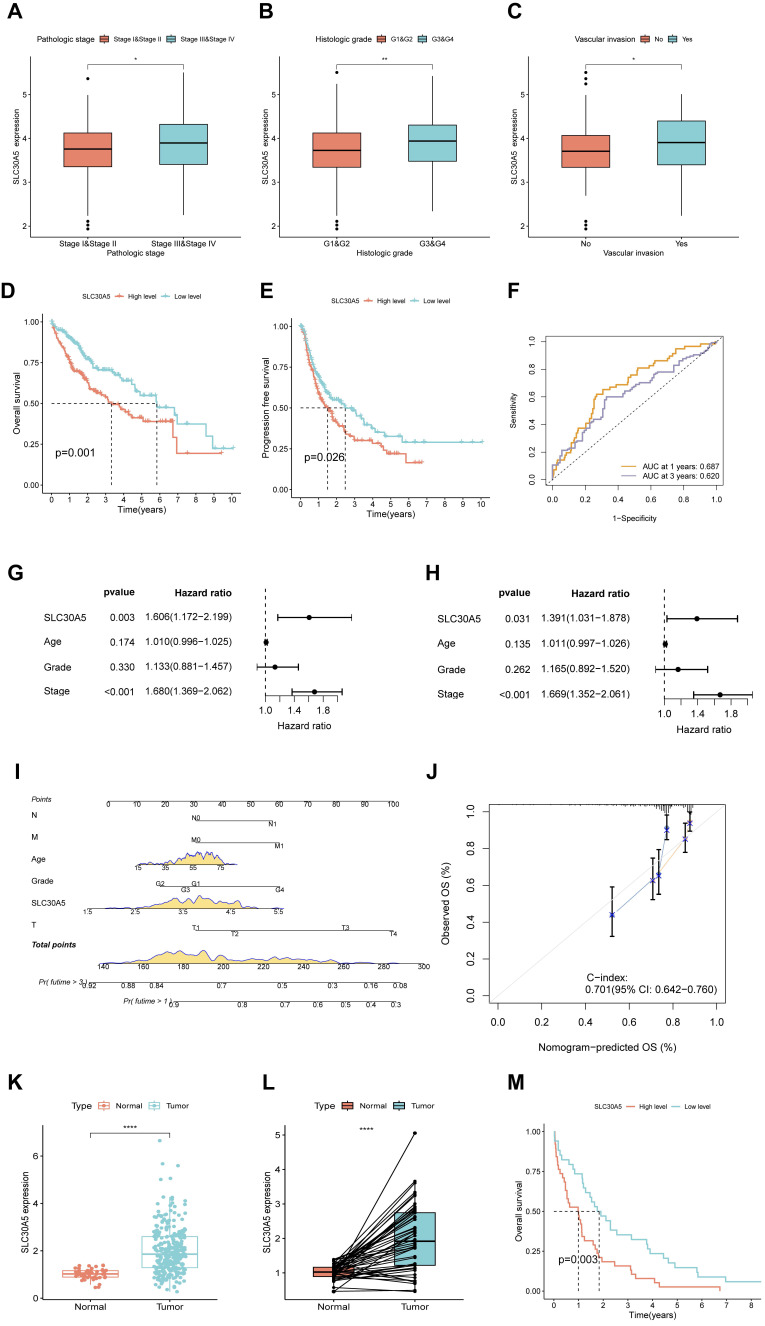
** Expression of SLC30A5 in HCC patients and its clinical relevance. (A)** Variation in SLC30A5 expression across different pathological stages; **(B)** Changes in SLC30A5 expression across different histological grades; **(C)** Association of SLC30A5 expression with vascular invasion;** (D, E)** Kaplan-Meier analysis illustrating the impact of SLC30A5 expression on OS (D) and PFS (E);** (F)** ROC curve for the prognostic evaluation of 1-year and 3-year OS in HCC patients based on SLC30A5 expression; **(G, H)** Univariate Cox analysis (G) and multivariate Cox analysis (H) revealing the prognostic significance of SLC30A5 in HCC; **(I)** Nomogram based on SLC30A5 expression, age, gender, and pathological stage; **(J)** Calibration plot evaluating the accuracy of the nomogram predictions; **(K)** SLC30A5 expression levels in the ICGC cohort; **(L)** Paired analysis of SLC30A5 expression levels between tumor and adjacent normal liver tissues in the ICGC cohort; **(M)** Kaplan-Meier survival curve correlating SLC30A5 expression in the ICGC cohort with overall survival. Statistical significance is denoted as follows: *p<0.05, **p<0.01, ***p<0.001, and ****p<0.0001.

Further investigation into the clinical correlation of SLC30A5 in HCC revealed that SLC30A5 expression was significantly elevated in patients with advanced pathological stages (Figure 3A), higher histologic grades (Figure 3B), and the occurrence of vascular invasion (Figure 3C). These findings suggested a potential association between the elevated expression of SLC30A5 and the aggressive progression of HCC.

Kaplan-Meier survival analysis indicated that patients with high SLC30A5 expression exhibited significantly poorer overall survival (OS) and progression-free survival (PFS) (Figure 3D-E). ROC analysis demonstrated the prognostic value of SLC30A5 in HCC, with AUC values for 1-year and 3-year survival predictions being 0.687 and 0.620, respectively (Figure 3F). SLC30A5 was confirmed as an independent prognostic marker for HCC through both univariate and multivariate Cox regression analyses (Figure 3G-H). To illustrate the clinical applicability of SLC30A5, a nomogram was constructed incorporating SLC30A5 expression along with patient age, gender, and pathological stage (Figure 3I). The predictive accuracy of the model was assessed by calibration curves, with a concordance index (C-index) of 0.701 (Figure 3J).

These results were validated using the ICGC-LIHC-US cohort, where both unpaired (Figure 3K) and paired comparisons (Figure 3L) consistently demonstrated elevated expression of SLC30A5 in HCC tissues. Additionally, Kaplan-Meier curve analysis showed significantly lower survival rates for HCC patients with high SLC30A5 expression within the ICGC cohort (Figure 3M).

### The role of SLC30A5 in the hepatocellular carcinoma tumor microenvironment

**Figure 4:**
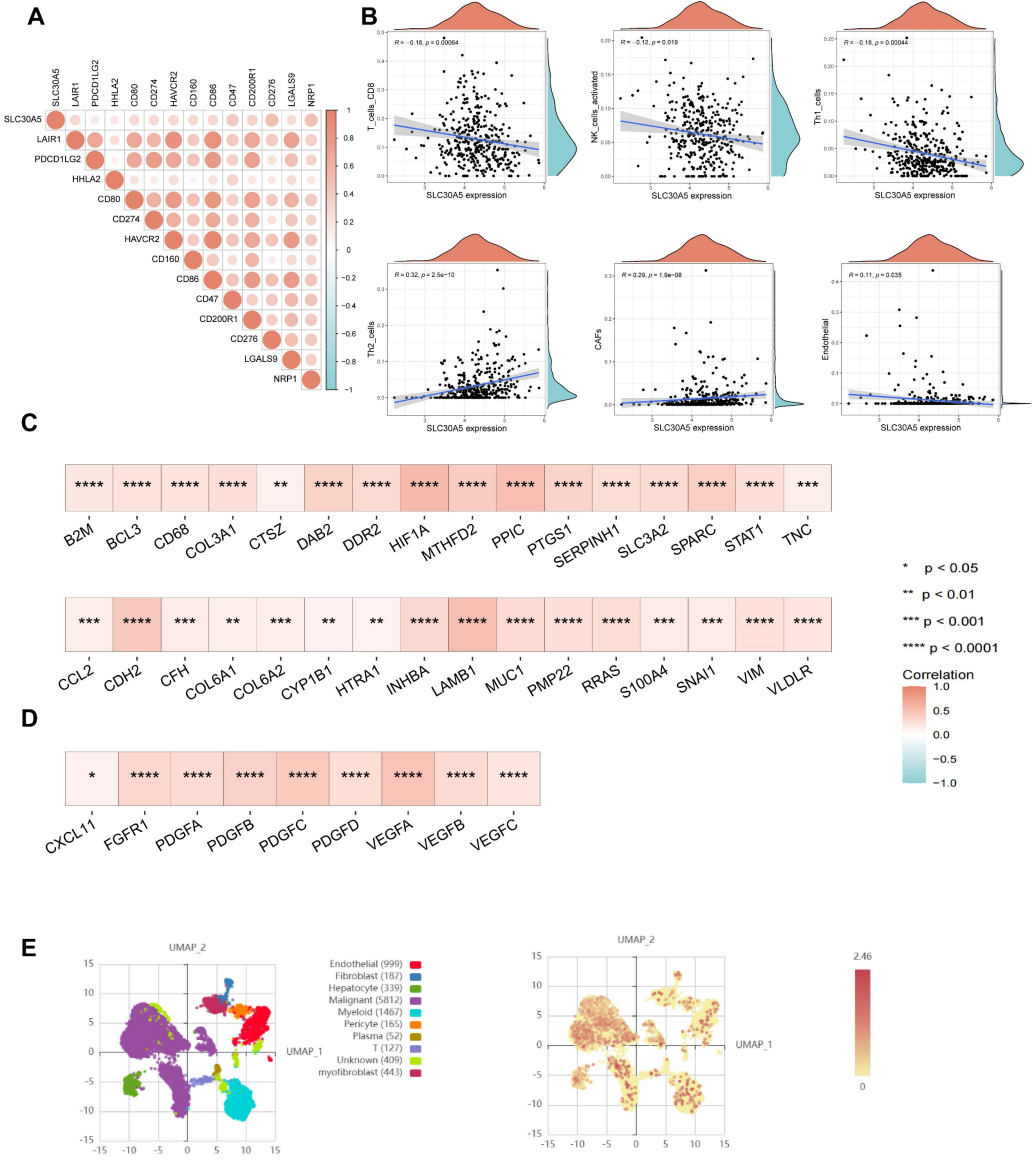
** The impact of SLC30A5 on the tumor microenvironment of HCC. (A)** Correlation between SLC30A5 expression and immune checkpoints;** (B)** Relationship between SLC30A5 expression and immune cell infiltration; **(C)** Correlation of SLC30A5 with molecules related to tumor angiogenesis; **(D)** Association of SLC30A5 with EMT-related molecules; **(E)** Single-cell sequencing analysis of SLC30A5 expression across different cell types in HCC.

The tumor microenvironment (TME) plays a crucial role in influencing tumor behavior, including proliferation, invasion, migration, and response to treatment 12. Cancer cells often manipulate immune checkpoints within the TME to evade immune surveillance and promote immune evasion. In this study, a positive correlation was observed between SLC30A5 expression and immune checkpoints, including CD274, CD276, PDCD1LG2, and HHLA2 (Figure 4A). Based on the TIMER database, SLC30A5 was associated with an immunosuppressive TME, characterized by a reduction in immune effector cells such as CD^8+^ T cells and NK cells. Additionally, SLC30A5 showed a negative correlation with anti-tumorigenic CD^4+^ Th1 cells and a positive correlation with pro-tumorigenic CD^4+^ Th2 cells 13 (Figure 4B).

Given the pronounced vascularization characteristic of HCC, the progression, invasion, and metastasis of HCC are closely linked to angiogenic processes and the formation of abnormal vascular structures 14. In this study, the overexpression of SLC30A5 correlated with vascular invasion in HCC patients, indicating its potential involvement in angiogenesis. Meanwhile, SLC30A5 was significantly positively correlated with cancer-associated fibroblasts (CAFs) and endothelial cells (Figure 4B), both of which play crucial roles in angiogenesis, proliferation and migration in HCC 15, 16. Additionally, SLC30A5 showed a significant positive correlation with pathway molecules related to tumor angiogenesis (Figure 4C) and EMT (Figure 4D).

At the single-cell level, analysis of the GSE112271 dataset investigated SLC30A5 expression across various HCC-associated cell types. Nine cell types were identified, including epithelial cells, fibroblasts, hepatocytes, malignant cells, and myeloid cells, among others. Predominant expression of SLC30A5 was observed in malignant cells (Figure 4E), highlighting its central involvement in driving HCC progression.

**Figure 5:**
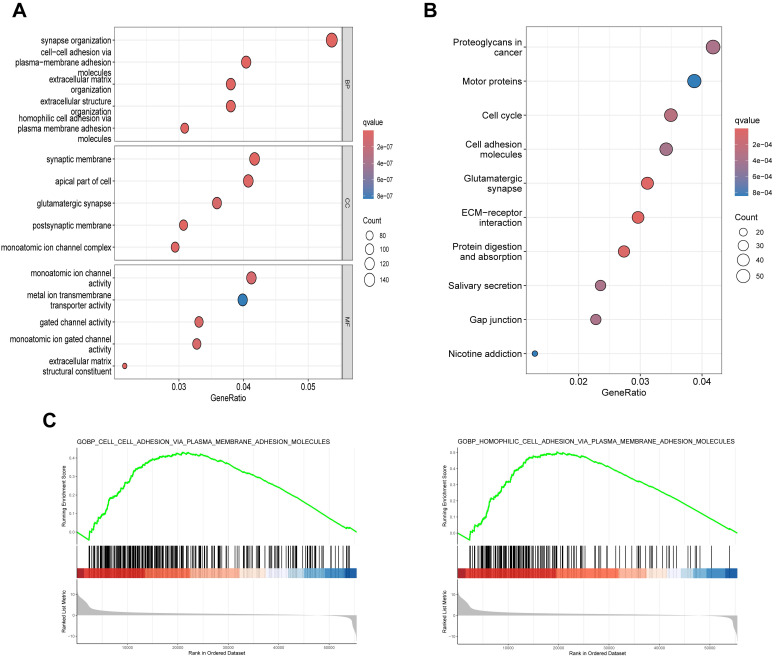
** Potential functions of SLC30A5 in HCC. (A, B)** GO (A) and KEGG (B) functional enrichment analyses of DEGs associated with SLC30A5; **(C)** GSEA enrichment analysis of SLC30A5.

To elucidate the biological functions of SLC30A5 in HCC, differentially expressed genes (DEGs) analysis was performed based on SLC30A5 expression (Supplementary Figure S7), followed by functional enrichment analyses. GO analysis revealed that these genes were primarily involved in cell adhesion and extracellular matrix (ECM) organization (Figure 5A). KEGG analysis showed that these molecules mainly participated in several cancer-related pathways, including those related to proteoglycans, molecular motors, the cell cycle, and interactions between cells and ECM receptors (Figure 5B). GSEA analysis further supported these findings, indicating that the cohort with high SLC30A5 expression was significantly enriched in cell adhesion pathways (Figure 5C).

### Tumor-promotive effects of SLC30A5 in hepatocellular carcinoma cells

**Figure 6:**
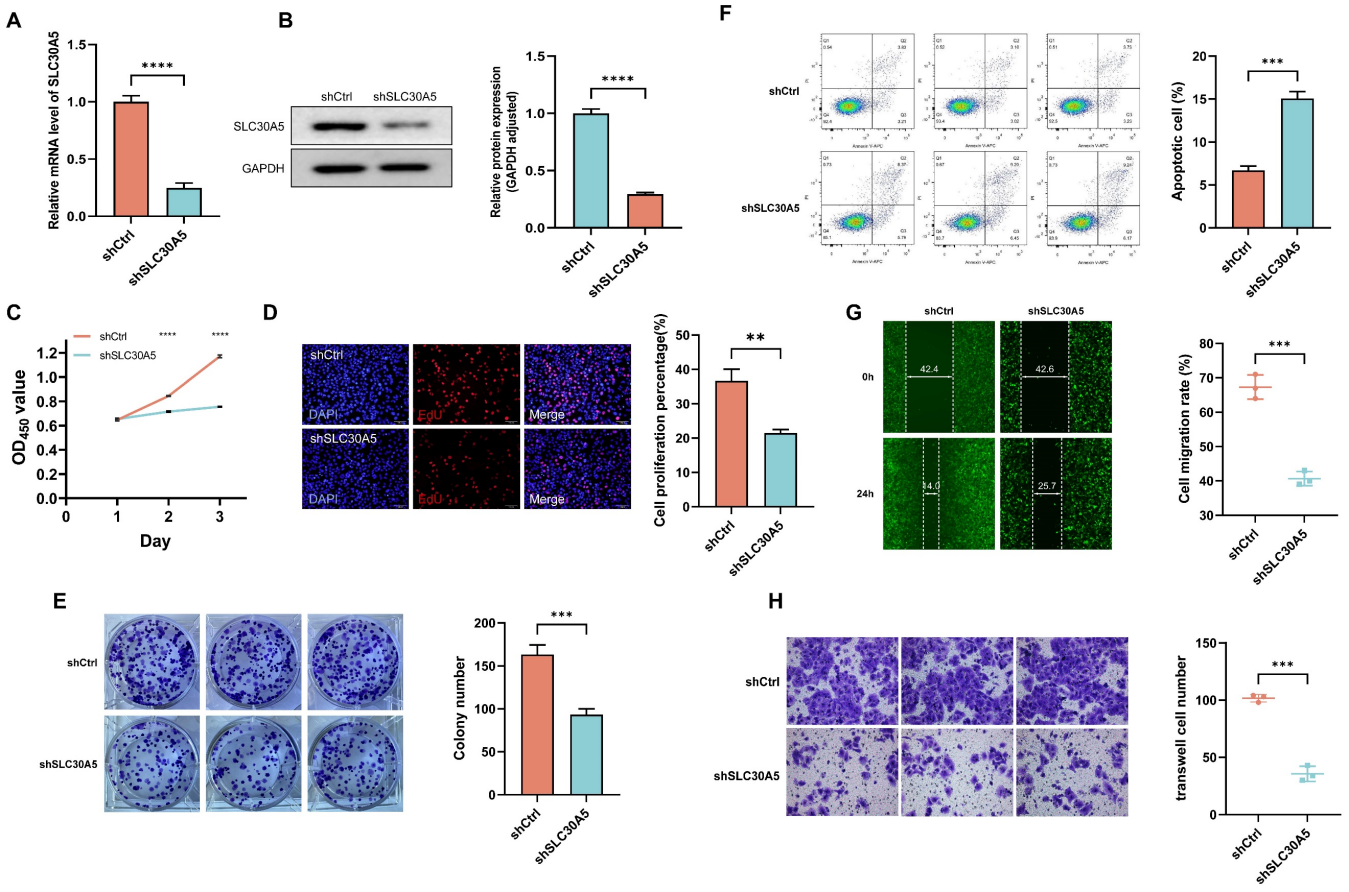
** The impact of SLC30A5 knockdown on hepatocellular carcinoma cells functions. (A, B)** Real-time PCR (A) and Western blot assays (B) verify the knockdown efficiency of SLC30A5 in Huh7 cells, n=3 in each batches; **(C-E)** The effects of SLC30A5 silencing on cell proliferation were evaluated using CCK-8 assays (C), EdU assays (D), and colony formation assays (E), n=3; **(F)** Apoptotic changes following SLC30A5 knockdown were quantified by flow cytometry, n=3; **(G, H)** The influence of SLC30A5 suppression on cell migration and invasion was assessed using wound healing assays (×100 magnification) (G) and Transwell invasion assays (×200 magnification) (H), n=3. Statistical significance is denoted as follows: *p<0.05, **p<0.01, ***p<0.001, and ****p<0.0001.

To explore the tumor-promoting effects of SLC30A5 in HCC cells, an shRNA vector targeting SLC30A5 was constructed and transfected into Huh7 cells. The knockdown efficiency was validated at both mRNA and protein levels through RT-PCR (Figure 6A) and Western blot analysis (Figure 6B), respectively. The impact of SLC30A5 suppression on cell viability was assessed using the CCK-8 assay, revealing a significant reduction in proliferation in the shSLC30A5 group compared to the control group (shCtrl) at various time points (Figure 6C). Furthermore, a significant decrease was observed in the percentage of EdU positive cells (Figure 6D) and colony formation in the SLC30A5 knockdown cells (Figure 6E). Meanwhile, the SLC30A5 knockdown group induced cell apoptosis (Figure 6F).

Moreover, wound healing assays demonstrated that SLC30A5 downregulation markedly impaired the migratory abilities of Huh7 cells (Figure 6G). Consistent with these findings, Transwell invasion assays showed that SLC30A5 knockdown significantly reduced the invasive potential of Huh7 cells (Figure 6H). Taken together, these findings illuminate the pivotal function of SLC30A5 in driving tumorigenesis processes in HCC through its modulation of cellular proliferation, apoptosis, migration, and invasion.

### Knockdown of SLC30A5 inhibits the growth of HCC *in vivo*

**Figure 7:**
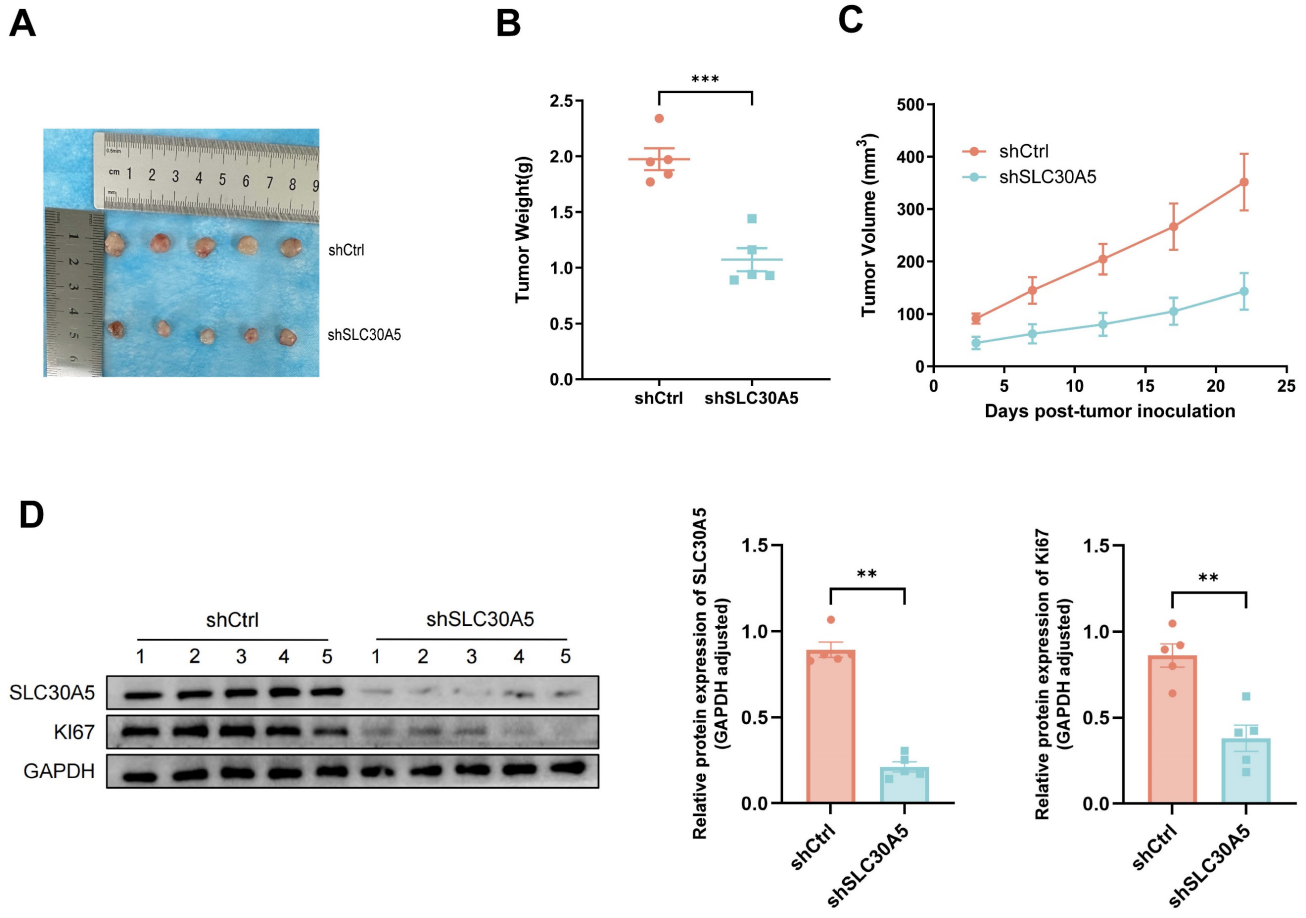
** SLC30A5 knockdown significantly reduced growth of HCC xenograft tumors in nude mice. (A)** Representative images of five xenograft tumors in nude mice;** (B)** Weight of dissected tumors, n=5; **(C)** Volume of tumor growth, n=5; **(D)** Expression level of SLC30A5 and Ki-67 within the tumors examined by western blot, n=5. Statistical significance is denoted as follows: **p<0.01, ***p<0.001.

To investigate the role of SLC30A5 in HCC growth *in vivo,* a nude mice xenograft tumor model was constructed. Huh7 cells with SLC30A5 knockdown and their corresponding negative control cells were subcutaneously inoculated into the mice. The findings revealed that tumors derived from the SLC30A5 knockdown cells were significantly smaller in size, weight, and volume compared to the controls (Figure 7A-C).

Further analysis demonstrated that tumors from the control group exhibited elevated expression levels of SLC30A5 and the proliferation marker Ki-67 compared to the SLC30A5 knockdown group (Figure 7D).

## Discussion

This study presents two key findings: Firstly, it investigated the role of SLC30A family genes in pan-cancer, revealing a notable correlation with cancer diagnosis and prognosis, particularly identifying SLC30A5 as a promising biomarker for HCC. Secondly, elevated expression of SLC30A5 is associated with unfavorable outcomes in HCC, and its overexpression promotes the proliferation, migration, and invasion of HCC cells.

Previous studies have highlighted the pivotal role of zinc transporter proteins in various cancers, including prostate, breast, and pancreatic cancers 6-9. Building upon this foundation, the present study conducted a comprehensive bioinformatics analysis alongside a series of *in vitro* and *in vivo* experiments to elucidate the potential role of SLC30A5 in the progression of HCC. The present study extends the current understanding of zinc transporter proteins in oncology, demonstrating the overexpression of SLC30A1, SLC30A5, SLC30A6, SLC30A7, and SLC30A9 in pan-cancer. This overexpression correlates with the prognosis of multiple cancers, suggesting their involvement in modulating tumor-related physiological and pathological processes 17. Among these, SLC30A5 was identified as potentially influential in HCC, warranting further investigation into its specific functions and mechanisms.

In this study, SLC30A5 was overexpressed in various tumors compared to normal tissues at the mRNA, protein, and cellular levels. The elevated expression of SLC30A5 in HCC is associated with adverse clinical outcomes. Further analyses demonstrated a significant inverse relationship between SLC30A5 expression and its methylation patterns, with notably reduced methylation in HCC. This finding suggests that an epigenetic mechanism may underlie the overexpression of SLC30A5. Furthermore, a positive correlation was identified between SLC30A5 and other members of the SLC30A gene family, such as SLC30A6 and SLC30A7, indicating an important role of SLC30A5 within the gene family. This relationship may also affect the expression of related genes, underscoring the potential role of SLC30A5 in the gene family and its impact on relevant biological pathways.

SLC30A5 is essential for the activation of ATX and MMP9, enzymes critical for cancer progression and metastasis 18. In the present study, experiments conducted both *in vivo* and *in vitro* revealed that knocking down SLC30A5 significantly reduced cell viability and proliferation, confirming its pivotal role in HCC cell growth. Furthermore, immune cell infiltration in the TME is crucial for modulating immune evasion and facilitating tumor progression 19. The present study demonstrated a negative correlation between SLC30A5 expression and the infiltration of CD^8+^ T cells and NK cells, which are key components of the anti-tumor immune response. This finding is consistent with existing literature, demonstrating a positive association between higher CD^8+^ T cell infiltration and favorable survival outcomes in HCC patients 20. Additionally, SLC30A5 expression was negatively correlated with CD^4+^ Th1 cells and positively correlated with CD^4+^ Th2 cell infiltration. Th2 cells are known to promote tumor growth and metastasis by secreting cytokines such as IL-4, IL-5, and IL-13 21, 22. The binding of IL-4 with its receptor on immune cells leads to the phosphorylation of STAT6, and the overexpression of STAT6 in the TME acts as an immunosuppressive signal, facilitating tumor cell proliferation 23, 24. Based on these findings, it is hypothesized that SLC30A5 may promote tumor proliferation by modulating the infiltration of immunosuppressive cells.

HCC frequently causes both intrahepatic and extrahepatic metastases 25. This study demonstrates that the knockdown of SLC30A5 significantly inhibits the migration and invasion of Huh7 cells, suggesting a potentially role in regulating tumor metastasis. The invasion and metastasis of tumors largely depend on their complex tissue environment 26. In the context of HCC, the TME characterized by aberrant angiogenesis and ECM remodeling, generates an immunosuppressive microenvironment that facilitates HCC proliferation and metastasis 27, 28.

In the current study, gene function enrichment analysis demonstrated the role of SLC30A5 in modulating cell adhesion, proteoglycans, and ECM receptor interactions. As integral components of the ECM, proteoglycans modulate cellular behaviors by interacting with various cytokines, growth factors, and adhesion molecules within the ECM. These interactions are crucial for tumor angiogenesis, proliferation, invasion, and metastasis 29, 30. This suggests that SLC30A5 may influence the metastasis of HCC through its role in cell adhesion and interactions with ECM components.

CAFs are among the most enriched components within the TME of HCC, playing a crucial role in driving metastasis and invasion processes 31. Recent study has reported that CAFs bolster immunosuppression and facilitate invasion by promoting myeloid-derived suppressor cell (MDSC) generation through the IL6/STAT3 pathway and SDF-1α 32. The present study identifies a significant positive correlation between SLC30A5 expression and CAFs, providing insight into the potential role of SLC30A5 in HCC metastasis.

Given the highly vascular nature of HCC, angiogenesis is critical to its growth and spread. Additionally, anti-angiogenic therapy is essential in managing advanced stages of HCC. In the present study, the positive correlation between SLC30A5 and molecules involved in tumor angiogenesis and EMT suggests a potential role in regulating tumor metastasis through these pathways. Furthermore, this correlation suggests that SLC30A5 may be a therapeutic target in anti-angiogenic therapy.

In summary, this study provides significant insights into the potential role of SLC30A5 in HCC metastasis, particularly through its interactions with the TME, cell adhesion mechanisms, and angiogenic pathways. While SLC30A9 is identified as an oncogene in colorectal cancer, potentially promoting tumor progression through the Wnt signaling pathway 5, the detailed mechanisms underlying SLC30A5-driven tumor metastasis, particularly in HCC, still await validation through *in vitro* or *in vivo* studies.

The link between SLC30A5 overexpression and adverse outcomes in HCC, along with its involvement in tumor proliferation and metastasis through immunosuppression, angiogenesis, and EMT, highlights SLC30A5 as a potential prognostic biomarker and therapeutic target in HCC. Beyond HCC, elevated levels of SLC30A5 have also been observed in cholangiocarcinoma (CHOL) and lung adenocarcinoma (LUAD), suggesting its broader impact and prognostic value across different types of cancer. Additionally, the distinct expression patterns of SLC30A family genes across various cancers, particularly their lower expression levels in renal cancers (KIRP, KIRC, KICH), underscore the potential importance of this gene family in oncology.

While this study provides preliminary insights into the role of SLC30A5 in HCC, it remains uncertain whether SLC30A5 exhibits carcinogenic potential in normal cells. Future research could further investigate this aspect. Additionally, assessing the role of SLC30A5 across different subtypes of HCC and exploring its potential as a personalized therapeutic target would be of considerable research value.

## Conclusion

In conclusion, this study elucidated the expression profile, prognostic significance, functional interactions, and immune infiltration of SLC30A5 in HCC. The findings indicate that SLC30A5 is overexpressed in HCC, which is associated with tumor proliferation and metastasis. SLC30A5 emerges as a promising candidate for a prognostic marker and a therapeutic target in HCC.

## Supplementary Material

Supplementary figures and table.

## Figures and Tables

**Table 1:** Association of high expression of SLC30A family genes with pan-cancer prognosis in different databases.

		TCGA (Kaplan-Meier)	TCGA (COX)	Kaplan-Meier plotter
SLC30A1	Protective	**KIRC, UCEC**	**KIRC, UCEC**	**KIRC,** OV, UCEC
	Detrimental	ACC, CESC, PAAD, LUSC	ACC, PAAD	CESC, KIRP, LUAD, LUSC, PAAD, READ
SLC30A2	Protective	ACC, KIRC	ACC	KIRC
	Detrimental	SKCM, UCEC, UVM	SARC, UCEC, UVM	BRCA, HNSC, LIHC, LUSC, PCPG, UCEC
SLC30A3	Protective	LAML	LAML	BRCA, LUSC, THCA
	Detrimental	BLCA, COAD, KIRC, KIRP, MESO	**KIRP**, UCEC, COAD, MESO, THYM, LIHC, ESCA, DLBC	BLCA, KIRC, KIRP, LIHC, STAD, THYM
SLC30A4	Protective	**KIRC**, PAAD, SARC	**KIRC**, PAAD	HNSC, KIRC, LUSC, PAAD, READ
	Detrimental		BLCA, THYM	BLCA, STAD, THYM, THCA
SLC30A5	Protective		KIRC	KIRC, PAAD, READ
	Detrimental	LGG, LIHC, READ	LGG, KIRP, LIHC, BRCA, KICH, PCPG	BRCA, KIRP, LIHC, PCPG, SARC, STAD, THCA
SLC30A6	Protective	COAD		KIRC
	Detrimental	ACC, LGG, LIHC, UCEC	LGG, UCEC, LIHC, KIRP, ACC, PAAD	CESC, KIRP, LIHC, LUAD, PAAD, PCPG, SARC, UCEC
SLC30A7	Protective	SKCM, OV	SKCM, LAML, OV	KIRC, READ, THYM
	Detrimental	ACC, LGG, LIHC	LGG, ACC, PAAD, SARC, UVM	LIHC, LUAD, SARC
SLC30A8	Protective	SKCM		
	Detrimental	**LIHC**	**LIHC**, HNSC, UCS	BLCA, CESC, KIRP, LIHC
SLC30A9	Protective	COAD,** KIRC**, KIRP, MESO	**KIRC**, KIRP, COAD	BLCA, KIRC, KIRP, UCEC
	Detrimental	THCA		BRCA, SARC, THCA
SLC30A10	Protective	LAML, OV	KICH	
	Detrimental	ACC, KIRC, ESCA	ACC, THYM, CESC, HNSC	BRCA, CESC, HNSC, KIRC, KIRP, PCPG, STAD

Note: Bold text indicates that high gene expression is significantly associated with OS in these cancers, and this correlation is consistent between different databases.
