# Supplementary material for: Integrated pan-cancer genomic analysis reveals the role of SLC30A5 in the proliferation, metastasis, and prognosis of hepatocellular carcinoma
Source: J Cancer. 2024 Jul 2;15(14):4686–99. doi: 10.7150/jca.97214 (PMC11242337; doi:10.7150/jca.97214)

Fig. S1 Box plots represent the expression levels of SLC30A family genes in tumors and normal tissues.

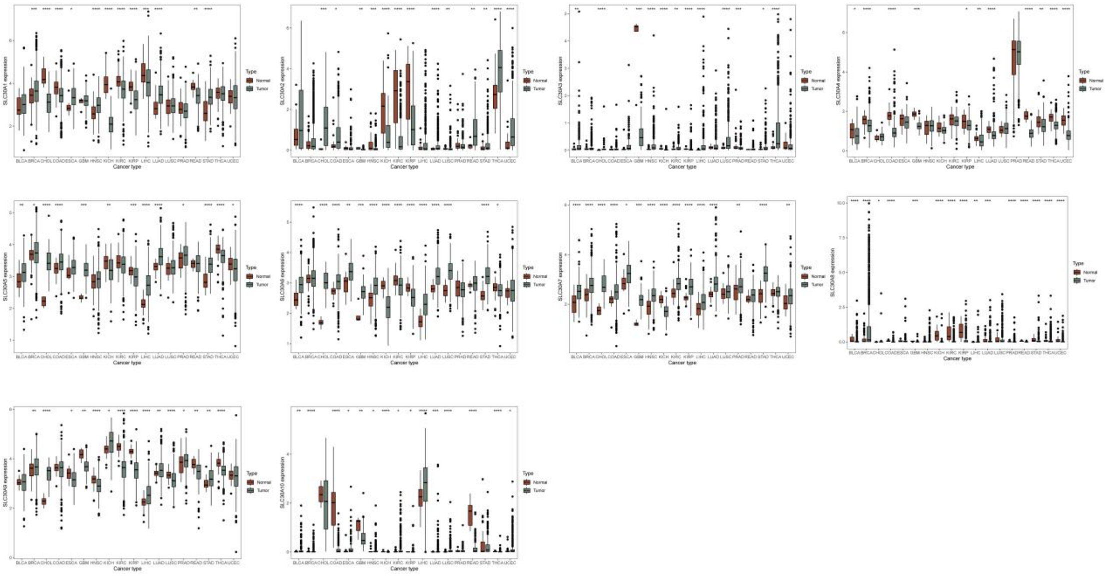

The bands in the box plot box are the median expression values of the genes. Statistical significance levels “*”, “**”, “***” and “****”) were indicated for P values < 0.05, < 0.01, < 0.001, and < 0.0001, respectively.

Fig.S2 Boxplot illustrating the expression levels of SLC30A family genes across seven types of cancer cell lines (breast, CNS/brain tumor, esophageal/gastric, renal, hepatocellular, lung, and pancreatic).

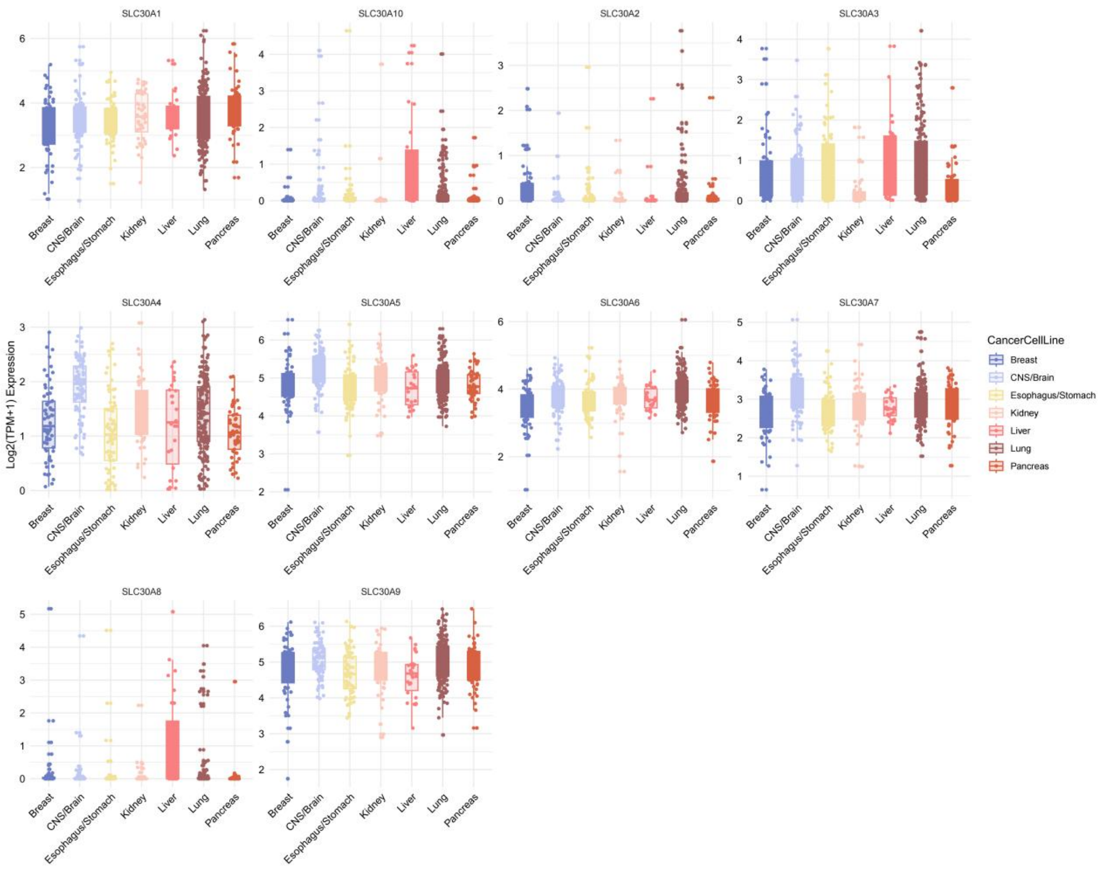

This data is sourced from the Cancer Cell Line Encyclopedia (CCLE) database.

Fig.S3 DNA Methylation of SLC30A family genes in pan-cancer.

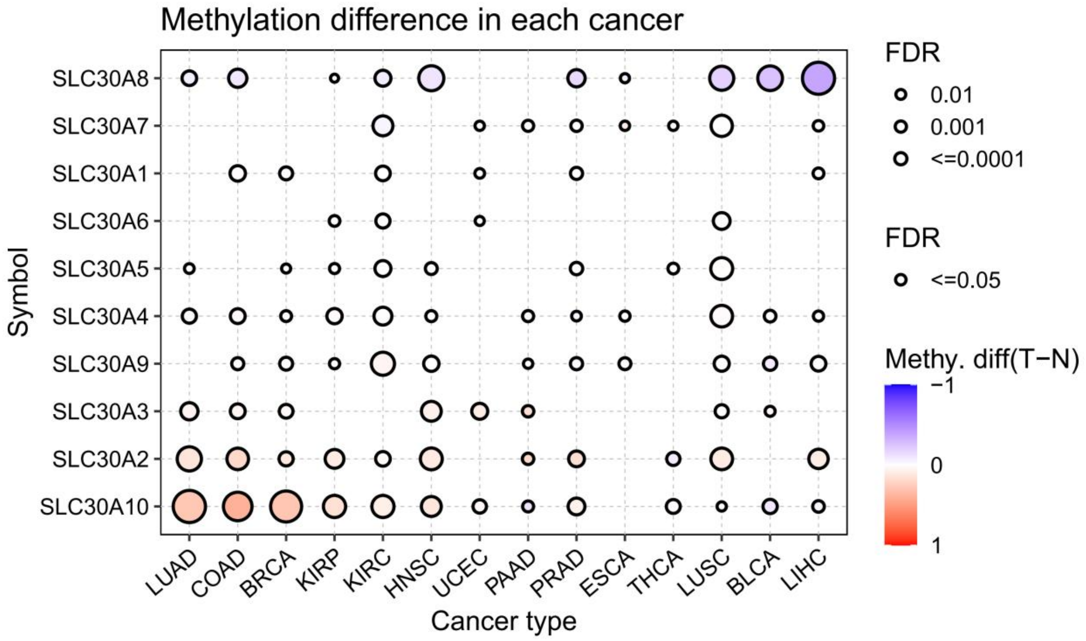

Fig.S4 Correlations between DNA methylation and mRNA expression of SLC30A family genes

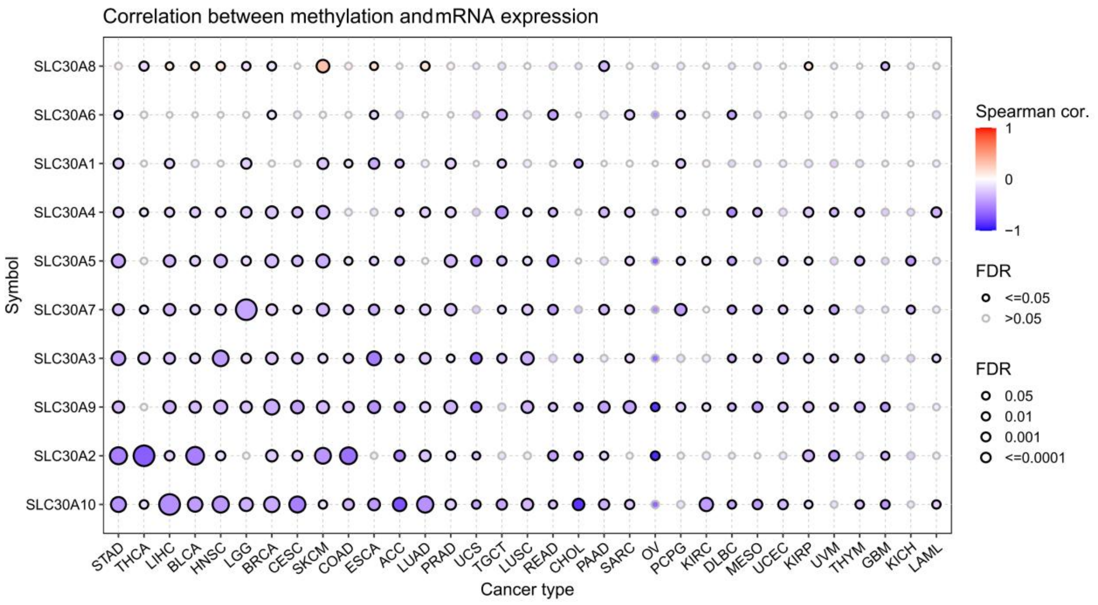

Fig.S5 Kaplan–Meier survival curve comparison of high/low expression of SLC30A family genes in pan-cancer.

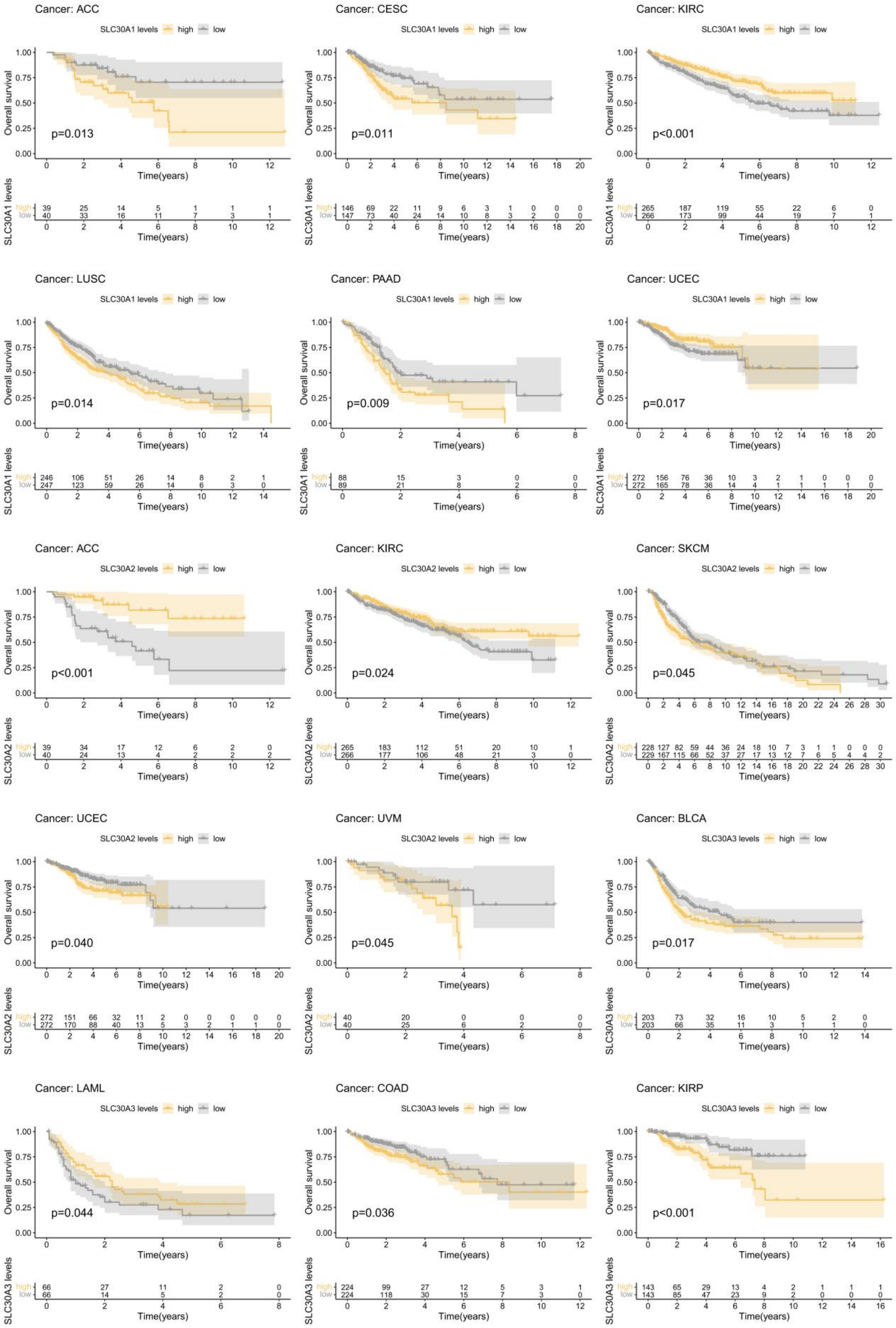

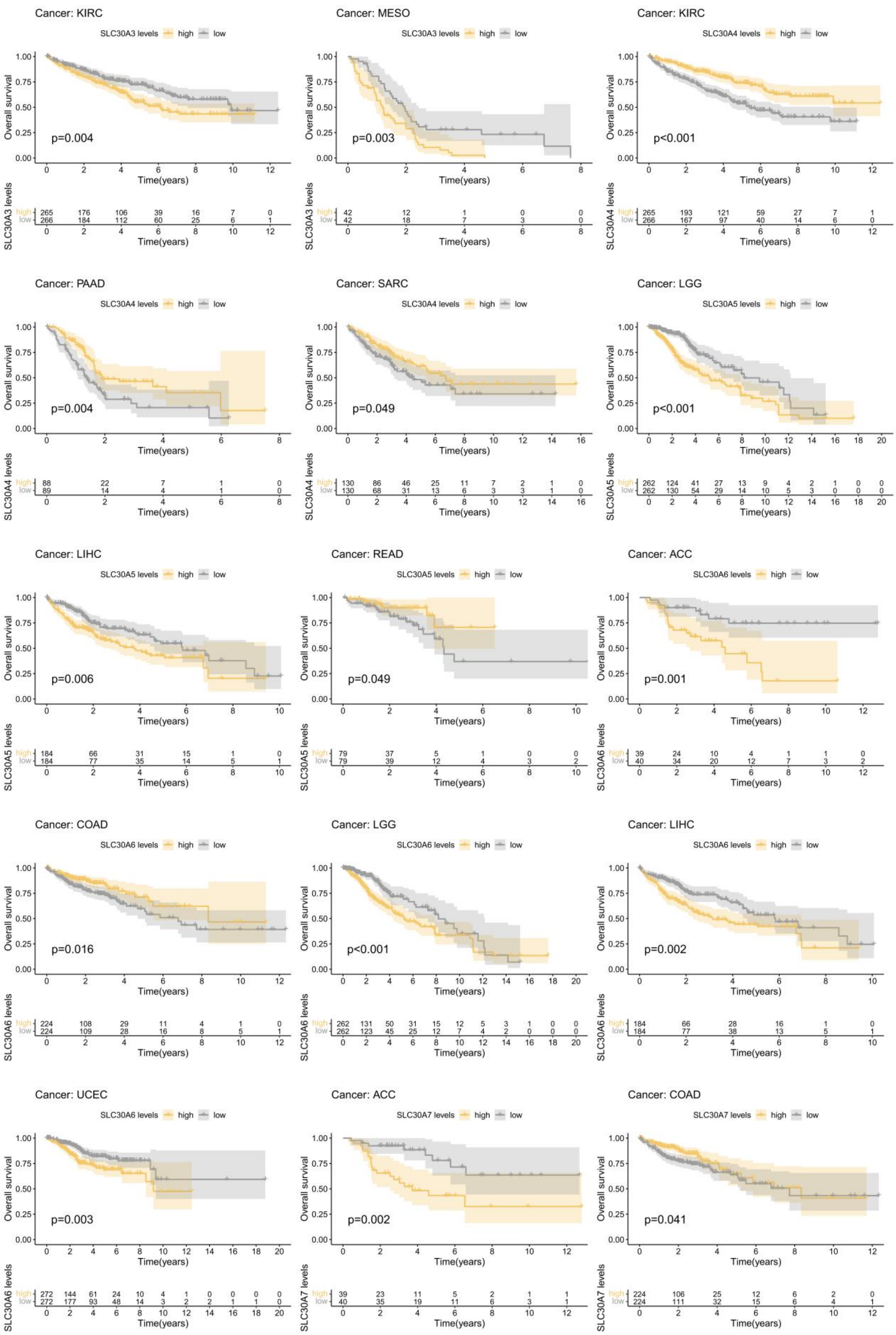

Cancer: KIRC
SLC30A3 levels high low
p=0.004
Cancer: MESO
SLC30A3 levels high low
p=0.003
Cancer: KIRC
SLC30A4 levels high low
p<0.001
Cancer: PAAD
SLC30A4 levels high low
p=0.004
Cancer: SARC
SLC30A4 levels high low
p=0.049
Cancer: LGG
SLC30A5 levels high low
p<0.001
Cancer: LIHC
SLC30A5 levels high low
p=0.006
Cancer: READ
SLC30A5 levels high low
p=0.049
Cancer: ACC
SLC30A6 levels high low
p=0.001
Cancer: COAD
SLC30A6 levels high low
p=0.016
Cancer: LGG
SLC30A6 levels high low
p<0.001
Cancer: LIHC
SLC30A6 levels high low
p=0.002
Cancer: UCEC
SLC30A6 levels high low
p=0.003
Cancer: ACC
SLC30A7 levels high low
p=0.002
Cancer: COAD
SLC30A7 levels high low
p=0.041
Overall survival
Time(years)

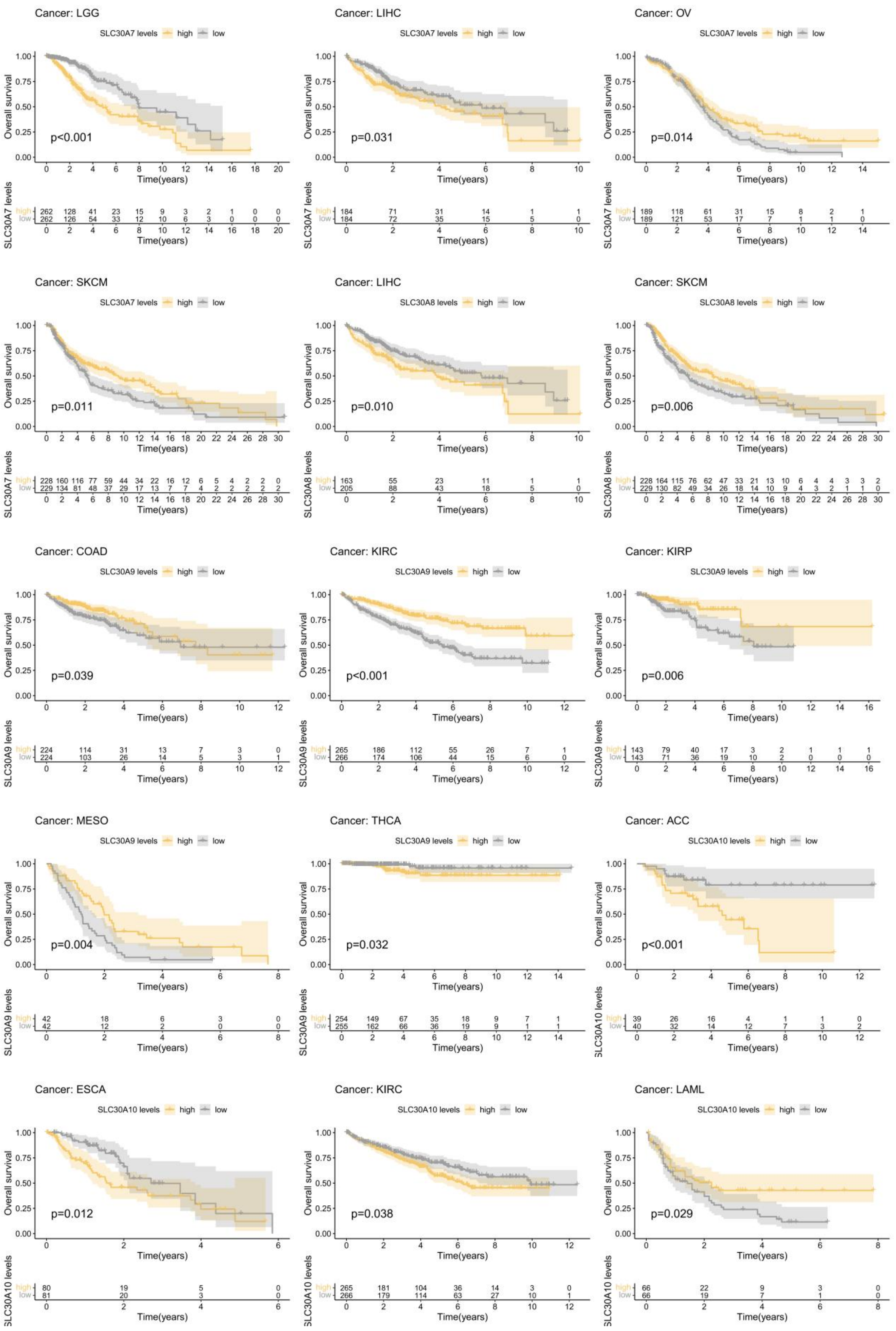
Cancer: LGG
SLC30A7 levels high low
p<0.001
Cancer: LIHC
SLC30A7 levels high low
p=0.031
Cancer: OV
SLC30A7 levels high low
p=0.014
Cancer: SKCM
SLC30A7 levels high low
p=0.011
Cancer: LIHC
SLC30A8 levels high low
p=0.010
Cancer: SKCM
SLC30A8 levels high low
p=0.006
Cancer: COAD
SLC30A9 levels high low
p=0.039
Cancer: KIRC
SLC30A9 levels high low
p<0.001
Cancer: KIRP
SLC30A9 levels high low
p=0.006
Cancer: MESO
SLC30A9 levels high low
p=0.004
Cancer: THCA
SLC30A9 levels high low
p=0.032
Cancer: ACC
SLC30A10 levels high low
p<0.001
Cancer: ESCA
SLC30A10 levels high low
p=0.012
Cancer: KIRC
SLC30A10 levels high low
p=0.038
Cancer: LAML
SLC30A10 levels high low
p=0.029
Overall survival
Time(years)

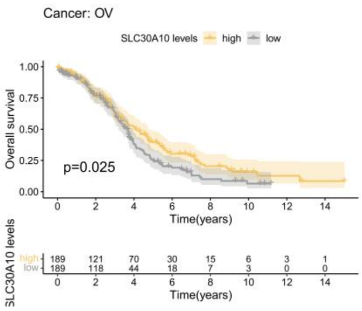

Cancer: OV
SLC30A10 levels high low
Overall survival
1.00
0.75
0.50
0.25
0.00
p=0.025
0 2 4 6 8 10 12 14
Time(years)
SLC30A10 levels
high 189 121 70 30 15 6 3 1
low 189 118 44 18 7 3 0 0
0 2 4 6 8 10 12 14
Time(years)

Fig.S6 Cox proportional hazard analysis indicates the overall survival rate associated with SLC30A fanmily genes expression in various cancer types.

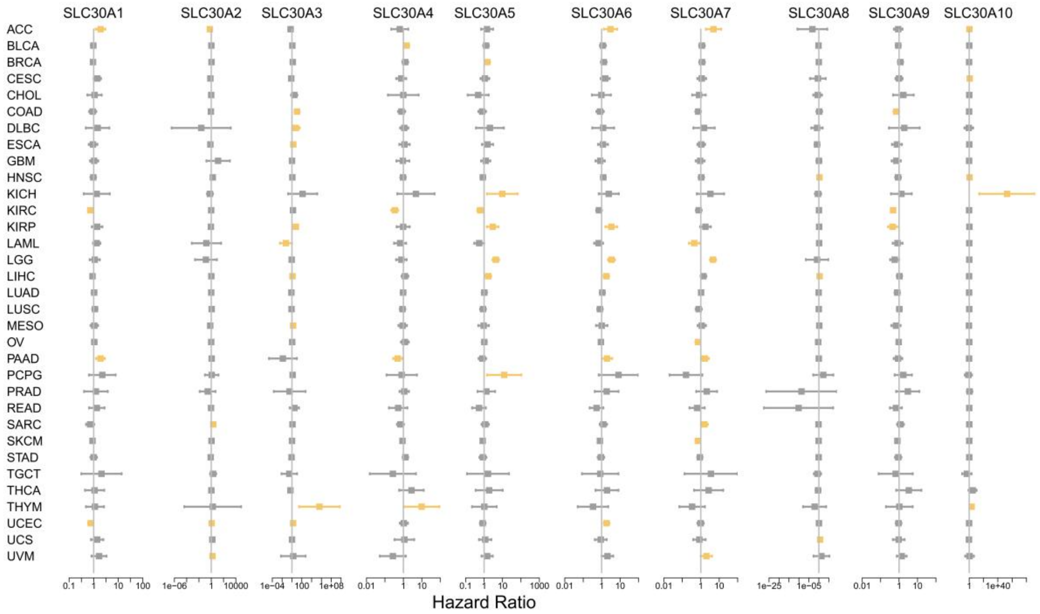

A hazard ratio <1 indicates low risk, and a hazard ratio >1 indicates high risk.

Fig.S7 Differential expression genes in high- and low-SLC30A5 expression groups.

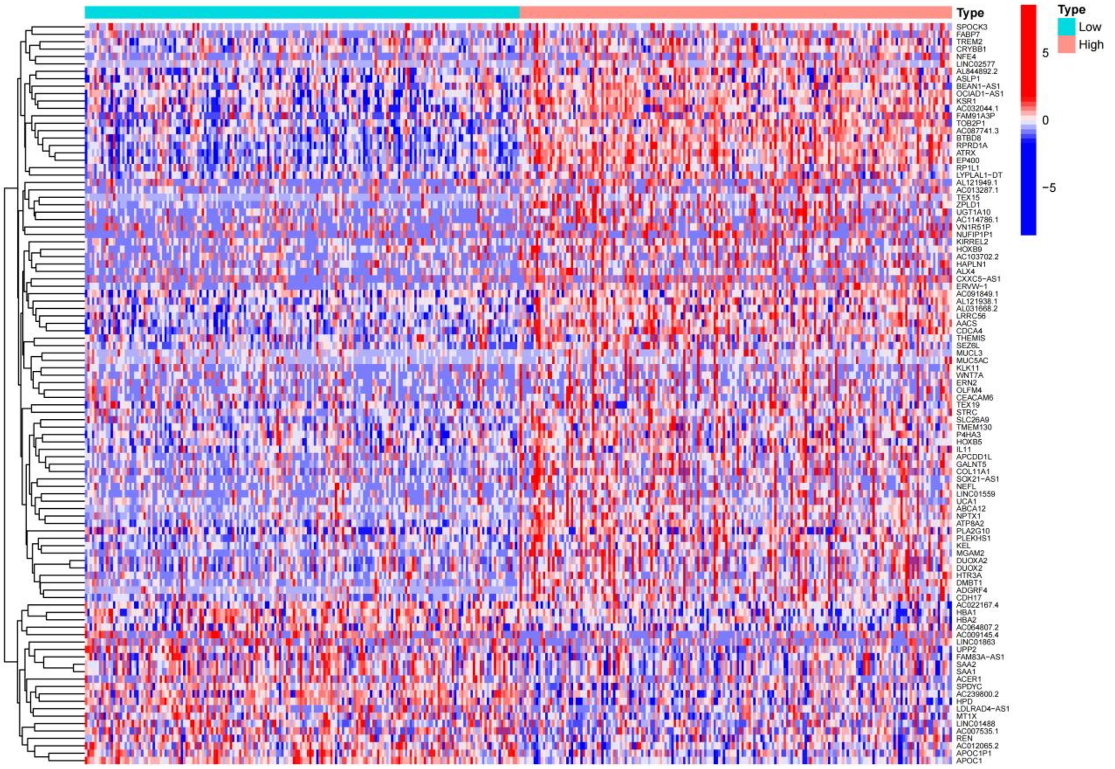

Supplement: Supplementary file 1 — Supplementary figures and table. [file jcav15p4686s1.zip › Supplementary Figure.pdf]
