# Supplementary material for: Integrated pan-cancer genomic analysis reveals the role of SLC30A5 in the proliferation, metastasis, and prognosis of hepatocellular carcinoma
Source: J Cancer. 2024 Jul 2;15(14):4686–99. doi: 10.7150/jca.97214 (PMC11242337; doi:10.7150/jca.97214)
Supplement: Supplementary file 1 — Supplementary figures and table. [file jcav15p4686s1.zip › Supplementary Table.pdf]

Table S1 Evaluation of SLC30A5, SLC30A6, and SLC30A9 Protein Expression Levels in 20 Cancer Types Using the Human Protein Atlas (HPA).

		Protein expression			Protein expression			Protein expression	
Cancer Type	Gene	High or Medium	Low or Not detected	Gene	High or Medium	Low or Not detected	Gene	High or Medium	Low or Not detected
glioma	SLC30A5	0.00%	100%	SLC30A6	20.00%	80.00%	SLC30A9	100.00%	0.00%
THCA	SLC30A5	100%	0.00%	SLC30A6	25.00%	75.00%	SLC30A9	100.00%	0.00%
LUCA	SLC30A5	25.00%	75.00%	SLC30A6	50.00%	50.00%	SLC30A9	100.00%	0.00%
COCA	SLC30A5	75.00%	25.00%	SLC30A6	30.00%	70.00%	SLC30A9	100.00%	0.00%
HNSC	SLC30A5	33.33%	66.67%	SLC30A6	50.00%	50.00%	SLC30A9	100.00%	0.00%
STCA	SLC30A5	20.00%	80.00%	SLC30A6	25.00%	75.00%	SLC30A9	100.00%	0.00%
LIHC	SLC30A5	58.33%	41.67%	SLC30A6	8.33%	91.67%	SLC30A9	100.00%	0.00%
carcinoid	SLC30A5	50.00%	50.00%	SLC30A6	25.00%	75.00%	SLC30A9	100.00%	0.00%
PACA	SLC30A5	90.00%	10.00%	SLC30A6	70.00%	30.00%	SLC30A9	100.00%	0.00%
RECA	SLC30A5	0.00%	100%	SLC30A6	100.00%	0.00%	SLC30A9	83.33%	16.67%
URCA	SLC30A5	10.00%	90.00%	SLC30A6	66.67%	33.33%	SLC30A9	100.00%	0.00%
PRCA	SLC30A5	33.33%	66.67%	SLC30A6	40.00%	60.00%	SLC30A9	100.00%	0.00%
TECA	SLC30A5	36.36%	63.64%	SLC30A6	50.00%	50.00%	SLC30A9	100.00%	0.00%
BRCA	SLC30A5	50.00%	50.00%	SLC30A6	45.45%	54.55%	SLC30A9	100.00%	0.00%
CECA	SLC30A5	27.27%	72.73%	SLC30A6	66.67%	33.33%	SLC30A9	100.00%	0.00%
ENCA	SLC30A5	40.00%	60.00%	SLC30A6	66.67%	33.33%	SLC30A9	100.00%	0.00%
OV	SLC30A5	50.00%	50.00%	SLC30A6	60.00%	40.00%	SLC30A9	100.00%	0.00%
melanoma	SLC30A5	27.27%	72.73%	SLC30A6	50.00%	50.00%	SLC30A9	100.00%	0.00%
SKCA	SLC30A5	25.00%	75.00%	SLC30A6	41.67%	58.33%	SLC30A9	100.00%	0.00%
lymphoma	SLC30A5	27.27%	72.73%	SLC30A6	16.67%	83.33%	SLC30A9	100.00%	0.00%
